# Longitudinal associations between stress and sleep disturbances during COVID‐19

**DOI:** 10.1002/smi.3144

**Published:** 2022-03-28

**Authors:** Andrea Ballesio, Andrea Zagaria, Alessandro Musetti, Vittorio Lenzo, Laura Palagini, Maria Catena Quattropani, Elena Vegni, Federica Bonazza, Maria Filosa, Tommaso Manari, Maria Francesca Freda, Emanuela Saita, Gianluca Castelnuovo, Giuseppe Plazzi, Caterina Lombardo, Christian Franceschini

**Affiliations:** ^1^ Department of Psychology Sapienza University of Rome Rome Italy; ^2^ Department of Humanities, Social Sciences and Cultural Industries University of Parma Parma Italy; ^3^ Department of Social and Educational Sciences of the Mediterranean Area University for Foreigners “Dante Alighieri” Reggio Calabria Italy; ^4^ Department of Clinical and Experimental Medicine, Psychiatric Section University of Pisa Azienda Ospedaliera Universitaria Pisana (AOUP) Pisa Italy; ^5^ Department of Educational Sciences University of Catania Catania Italy; ^6^ Department of Health Sciences University of Milan Milan Italy; ^7^ Department of Medicine and Surgery University of Parma Parma Italy; ^8^ Department of Humanistic Studies Federico II University Naples Italy; ^9^ Department of Psychology Catholic University of Milan Milan Italy; ^10^ Istituto Auxologico Italiano IRCCS Psychology Research Laboratory Verbania Italy; ^11^ Department of Biomedical, Metabolic and Neural Sciences University of Modena and Reggio Emilia Modena Italy; ^12^ IRCCS Istituto delle Scienze Neurologiche di Bologna Bologna Italy

**Keywords:** arousal, COVID‐19, HPA axis, mental health, pandemic, sleep, stress

## Abstract

The psychological consequences of COVID‐19 pandemic may include the activation of stress systems, that involve the hypothalamic‐pituitary‐adrenal axis which influences many physiological functions, including sleep. Despite epidemiological studies evidenced greater prevalence of stress symptoms and sleep disturbances during COVID‐19, longitudinal evidence investigating the effects of stress on sleep disturbances during the pandemic is lacking. We collected measures of perceived stress and sleep disturbances during the first wave of COVID‐19 (March 2020) and at 8–10 months follow up in a sample of 648 adults (*M* = 33.52, SD = 12.98 years). Results showed that 39.4% of participants reported moderate to extremely severe stress in March 2020. Prevalence of sleep disturbances was 54.8% in March 2020 and 57.4% at follow‐up. Structural equation modelling highlighted that perceived stress in March 2020 significantly predicted sleep disturbances at follow up (*β* = 0.203; *p* < 0.001), even after controlling for baseline sleep disturbances. Results remained significant even after controlling for the effects of covariates including age, sex, depression and anxiety symptoms, and referring to psychological services (*β* = 0.179; *p* < 0.05). Findings confirm the high prevalence of stress symptoms during the COVID‐19 pandemic and provide first longitudinal evidence for the effects of perceived stress on sleep disturbances during the pandemic.

## 1 INTRODUCTION

Since March 2020, COVID‐19‐related psychological research has profoundly investigated the effects of the pandemic and the consequent restrictions on cognitive‐behavioural and emotional variables. Within this context, stress is considered a major concomitant of the pandemic. In July 2020, a meta‐analysis on the prevalence of stress in the general population pooling the results from 14 individual studies highlighted that up to 24% of adult individuals suffered of post‐traumatic stress symptoms and 25% reported general stress symptoms (Cooke et al., 2020), which represent an increase considering that pre‐pandemic prevalence was estimated at 6%–7% (Pietrzaka et al., 2011). In Italy, the first European “hotspot” of COVID‐19 spreading, the percentage of adults reporting moderate to extreme levels of stress was estimated at 50% in the general population (Cellini et al., 2020).

The traditional neuroendocrine stress response involves the activation of the hypothalamic‐pituitary‐adrenal (HPA) axis which modulates many physiological functions, including sleep and circadian rhythm (Lo Martire et al., 2020). In fact, despite the great inter‐individual variability of stress response (identical stressors may elicit different reactions across individuals, Ellis et al., 2006), a frequently identified consequence of stress is sleep disturbance. In both animal and human models, the induction of chronic stress is associated with remarkable changes in sleep architecture [e.g., reduced slow wave (deep) sleep, reduced latency to the onset of the first rapid eye movement (REM) sleep period, and increased wakefulness], which are largely mediated by HPA hormones such as corticotropin releasing hormone, adrenocorticotropin hormone, and cortisol (Cheeta et al., 1997; Lo Martire et al., 2020). Moreover, within the framework of behavioural sleep medicine, etiological theories built upon the stress‐diathesis model (e.g., Perlis et al., 1997; Spielman et al., 1987) postulate the presence of stressful life events as a precipitating factor for the onset of sleep disturbances such as insomnia. Thus, it is plausible to hypothesize that the COVID‐19 event may have triggered sleep disturbances in already at‐risk individuals.

Several studies confirmed the presence of poorer or more disturbed sleep during COVID‐19 compared to pre‐pandemic period. For instance, Benham (2020) reported greater sleep onset latency (i.e., the time needed to fall asleep) and lower sleep efficiency (i.e., the ratio of total sleep time to time in bed) in Spring‐Summer 2020 compared to the same period of 2019. Saalwirth and Leipold (2021) reported poorer sleep quality during COVID‐19 pandemic compared to the previous period. With respect to clinical sleep outcomes, available studies overall demonstrated a greater prevalence of sleep disturbances ranging from 18% (Bacaro et al., 2020) to 42% (Gualano et al., 2020), compared to pre‐COVID‐19 estimates (e.g., 7%, Ohayon & Smirne, 2002).

### 1.1 Aim of the study

Despite the consensus regarding the conceptualization of COVID‐19 as a chronic stressful event (e.g., Saalwirth & Leipold, 2021; Taylor, 2021), the effects of perceived stress on psychophysiological clinical outcomes during the pandemic remain largely under‐researched. Specifically, the longitudinal associations between stress and sleep disturbances are yet to be clarified. In line with literature on the stress‐sleep relationship (see Lo Martire et al., 2020 for a review) and the etiological models explaining the role of stress in the onset of sleep disturbances (e.g., Spielman et al., 1987; Perlis et al., 1997), the aim of this study was to test the longitudinal associations between perceived stress and sleep disturbances at 8–10 months follow up using a structural equation modelling approach.

## 2 METHODS

### 2.1 Participants and procedure

The study was approved by the Ethics Committee of the Center for Research and Psychological Intervention (CERIP) of the University of Messina (n. 12106). All procedures performed were in accordance with the Declaration of Helsinki. A longitudinal survey design was implemented. The online survey was created through the Microsoft Azure platform. Prior to complete demographic information and self‐report questionnaires, a formal consent to process personal data was obtained for all participants involved in our study. Anonymity was guaranteed without collecting information that could identify the participants. Participants were recruited using numerous sources of publishing, such as university platforms, social networks, and online blogs. Only participants who lived in Italy were included in our study. The T1 data were collected during the Italian first pandemic wave, specifically from March 2020 to May 2020. The T2 data were collected during the Italian second pandemic wave, specifically from December 2020 to February 2021.

### 2.2 Measures

Socio‐demographic information such as sex, age, residence area, and marital status were investigated. Participants were also asked about their current or previous positivity to SARS‐CoV‐2 and about the death of a close contact due to COVID‐19.

The Depression Anxiety Stress Scale (DASS‐21; Lovibond & Lovibond, 1995; Bottesi et al., 2015) was employed to assess perceived stress and psychopathology symptoms. The DASS‐21 is a self‐reported 3‐factor instrument assessing depression (e.g., “I couldn't experience any positive feeling at all”), anxiety (e.g., “I was aware of the action of my heart in the absence of physical exertion”) and stress (e.g., “I felt that I was using a lot of nervous energy”) symptoms. Responses are coded on a 4‐point Likert scale ranging from 0 (never) to 3 (almost always), with higher scores indicating more severe symptoms. The stress scale was developed to assess commonly experienced stress symptoms including difficulty relaxing, nervous arousal, and being easily upset/agitated, irritable/over‐reactive and impatient (Lovibond & Lovibond, 1995). The DASS‐21 showed a stable and clear factor structure, that is, the three scales are factorially distinct from one another, as well as good psychometric properties in clinical and non‐clinical sample (e.g., Antony et al., 1998).

The Medical Outcome Study (MOS, Hays & Stewart, 1992) Sleep Scale was employed to assess the presence of sleep disturbances. The 12‐item scale assesses six sleep features during the previous month: sleep initiation, maintenance, respiratory problems, quantity, perceived adequacy, and somnolence. A sleep problem index can be calculated by aggregating the responses of the items, providing an overall measure of sleep disturbances, with higher scores in the composite scale indicate greater sleep disturbances. The scale showed robust psychometric properties in previous large representative samples (e.g., Hays et al., 2005). In our investigation, we considered the MOS sleep problem index as study outcome.

### 2.3 Data analysis

All data were analyzed using IBM SPSS version 23, and Mplus 8.6. Prior to model testing, univariate normality was checked for all items, where absolute skewness and kurtosis values greater than |1| reflect normality deviations (Marcoulides & Hershberger, 1997). Several items were above the cut‐off, suggesting realistically not normal distributions. Associations among constructs were evaluated using structural equation modelling (SEM) approach. More specifically, a full SEM was implemented hypothesizing a direct path from the DASS‐21 stress index measured during the first pandemic wave to self‐reported sleep disturbances measured during the second pandemic wave. A two‐step analysis was performed. In the first model, the effect was adjusted only for baseline sleep disturbances. In the second model, the other DASS‐21 subscales (i.e., anxiety and depression), demographic variables (i.e., age and sex), and COVID‐19 related variables (i.e., positivity to SARS‐CoV‐2, death of a close contact, and having attended psychiatric or psychological support services due to pandemic) were also inserted as covariates. A full SEM was composed of two primary components: the measurement model that specifies the relationships between observed variables and the underlying latent dimensions, and the structural model that specifies the relationship among the latent constructs (Weston & Gore, 2006). Before implementing the structural model, the adequacy of the related measurement model was tested through a confirmatory factorial approach defining the latent variables by their respective items as observed indicators (Bollen, 1989). The reliability of the measurement model was assessed using the McDonald's omega coefficients. Computations of omegas were based on the solution described in Hancock and An (2020). According to Bagozzi and Yi (2012), omega values greater than 0.70 were considered acceptable. The convergent validity of the measurement model was tested based on the magnitude of the standardized factor loadings and the average variance extracted (AVE) values (Hair et al., 2019). Factor loadings and AVE values greater than 0.5 are considered acceptable (Hair et al., 2019). Discriminant validity of the constructs' measures was assumed if correlations between latent constructs were not significantly larger than 0.85 (Kline, 2011). Following a multifaceted conception of model fit (Tanaka, 1993), several fit indices were reported: Root Mean Square Error of Approximation (RMSEA), Comparative Fit Index (CFI), Tucker‐Lewis Index (TLI) and the Standardized Root Mean Square Residual (SRMR). RMSEA values below 0.08, CFI values above 0.90, TLI values above 0.90, and SRMR values below 0.08 indicate an acceptable fit of the model to the data (Hu & Bentler, 1999). Chi‐square statistic was reported but not considered due to its sensitivity to large sample size (Cheung & Rensvold, 2002). Considering that DASS‐21 items have only four response options, we treated data as ordinal (option “categorical” in MPLUS) and the robust weighted least squares ‐ means and variance adjusted (WLSMV) method was used to estimate the model parameters (Muthén et al., 2002). To ensure the feasibility of our analysis and to assess whether sleep disturbances scale (MOS) was measuring the construct consistently over time, we performed longitudinal measurement invariance tests across the two pandemic waves. To compare these nested models, differences in CFI and RMSEA lower than 0.01 and 0.015, respectively, indicate a meaningless change in model fit (Chen, 2007; Cheung & Rensvold, 2002).

## 3 RESULTS

### 3.1 Sample characteristics

A total of 648 participants (*M*_age_ = 33.52, SD = 12.98 years; 78.7% females) were enroled. Baseline characteristics of the sample are reported in Table 1. Participants were predominantly resident in northern Italy (77.6%), the country area most impacted by the first wave of the pandemic. A substantial percentage of the participants were single (34%) and childless (74.5%). The 5.3% of the sample resulted positive to SARS‐CoV2 during the 3 months before the assessment, while 9.6% lost a loved one due to COVID‐19. A small number of participants (i.e., 15.3%) made use of psychological/psychiatric support services. The mean score in the stress scale was 16.32 (SD = 9.79). According to the DASS‐21 cut‐offs (Lovibond & Lovibond, 1995), 47.1% of participants were categorized as “normal”, 13.5% as “mild”, 18.7% as “moderate”, 15.6% as “severe”, 5.1 as “extremely severe” level of stress. The mean score at baseline of the sleep problem index was 31.50 (SD = 18.78), ranging from 0 to 97.22. The prevalence of sleep disturbances was 54.8% at baseline and increased at 57.4% at follow‐up.

**TABLE 1:** Baseline characteristics of the sample

Variable	M (SD) / %
Age	33.52 (12.98)
Sex (females)	78.7%
North Italy	77.6%
Central Italy	21.8%
South Italy	0.6%
Referral to psychological services	15.3%
Lost due to COVID‐19	9.6%
Positivity to SARS‐CoV2	5.3%
Depression (DASS‐21)	11.59 (5.9)
Anxiety (DASS‐21)	6.83 (7.1)
Stress (DASS‐21)	16.32 (9.79)
Sleep disturbances (MOS)	20.30 (4.28)

Abbreviations: DASS‐21, Depression, Anxiety, and Stress Scale; MOS, Medical Outcome Study Sleep Scale.

### 3.2 Measurement model

To test our measurement model, a CFA was firstly implemented positing the latent variables used in our investigation (i.e., stress, anxiety, depression, and sleep disturbances) as defined by their respective items as manifest indicators. Considering their longitudinal nature, the uniquenesses between the same MOS items across time were allowed to covariate (Little, 2013). Errors covariance between item 4 and item 12 of the MOS was also a priori freely estimated (i.e., the only reversed items). Mplus modification indices also suggested to covariate uniquenesses between item 1 and item 7 of the T2 MOS. This choice was theoretically supported by the narrowly similar content of the items that assess the same insomnia facet (i.e., sleep onset latency, SOL). The model showed a good fit to the data: *χ* ^2^ (680) = 2057.251, *p* < 0.001, CFI = 0.946, TLI = 0.941, RMSEA = 0.056 (90% CI = 0.053–0.059), SRMR = 0.055. We followed with two step, constraining factor loadings and items' thresholds of the MOS to be equal across time, respectively, establishing for metric invariance (CFI and RMSEA improved) and partial scalar invariance after relaxing thresholds of item 4 (ΔCFI = 0.003; ΔRMSEA = 0.001). Consistently, we maintained these constraints. This latter model showed a good fit to the data: *χ* ^2^ (723) = 2083.578, *p* < 0.001, CFI = 0.947, TLI = 0.945, RMSEA = 0.054 (90% CI = 0.051–0.057), SRMR = 0.056. Results are summarized in Table 2. In terms of reliability, all latent dimensions showed a greater omega value (*ω* ranged from 0.821 to 0.898). In terms of convergent validity, all factor loadings were statistically significant, ranging from 0.49 to 0.91, while AVE values were extremely close to or above the 0.5 cut‐off. On the other hand, correlations among latent constructs were all below 0.85, concluding there were no discriminant validity issues (Kline, 2011).

**TABLE 2:** Results of the measurement model

Measure	Item	Standardized factor loading^a^	Omega	AVE
Sleep problem index at baseline (MOS)	MOS1	0.730	0.849	0.49
	MOS3	0.798		
	MOS4 (reversed)	0.636		
	MOS5	0.702		
	MOS6	0.713		
	MOS7	0.825		
	MOS8	0.704		
	MOS9	0.604		
	MOS12 (reversed)	0.521		
Sleep problem index at follow‐up (MOS)	MOS1	0.717	0.859	0.47
	MOS3	0.783		
	MOS4 (reversed)	0.625		
	MOS5	0.690		
	MOS6	0.700		
	MOS7	0.810		
	MOS8	0.691		
	MOS9	0.593		
	MOS12 (reversed)	0.512		
Stress (DASS‐21)	DASS1	0.794	0.898	0.65
	DASS6	0.747		
	DASS8	0.766		
	DASS11	0.866		
	DASS12	0.917		
	DASS14	0.781		
	DASS18	0.773		
Anxiety (DASS‐21)	DASS2	0.491	0.821	0.54
	DASS4	0.735		
	DASS7	0.664		
	DASS9	0.800		
	DASS15	0.876		
	DASS19	0.720		
	DASS20	0.806		
Depression (DASS‐21)	DASS3	0.827	0.879	0.62
	DASS5	0.649		
	DASS10	0.790		
	DASS13	0.901		
	DASS16	0.798		
	DASS17	0.747		
	DASS21	0.807		

Abbreviations: AVE, Average Variance Extracted; DASS‐21, Depression, Anxiety, and Stress Scale; MOS, Medical Outcome Study Sleep Scale.

^a^ All factor loadings are statistically significant (*p* < 0.001).

### 3.3 Structural model

First, we implemented a model positing a direct effect from stress symptoms (DASS‐21) measured during the first pandemic wave to sleep disturbances (MOS) measured during the second pandemic wave, controlling for baseline sleep disturbances. The structural model exhibited an adequate fit to the data: χ^2^ (303) = 1273.595, *p* < 0.001, CFI = 0.945, TLI = 0.946, RMSEA = 0.070 (90% CI = 0.066–0.074), SRMR = 0.057. Findings showed that the stress prospectively predicted sleep disturbances (*β* = 0.203; *p* < 0.001).

Second, the analysis was also adjusted for the other DASS‐21 subscales (i.e., depression and anxiety), demographic variables (i.e., gender and age), and COVID‐19 related variables (i.e., positivity to SARS‐CoV2, death of a strict contact due to COVID‐19 and having attended psychological or psychiatric support services due to pandemic). The model showed a good fit to the data: *χ* ^2^ (913) = 2279.601, *p* < 0.001, CFI = 0.941, TLI = 0.940, RMSEA = 0.048 (90% CI = 0.046–0.051), SRMR = 0.078. The effect of stress symptoms on sleep disturbances was still significant (*β* = 0.179, *p* < 0.05). Regarding the covariates, sex (i.e., codified as a dummy variable where 0 = male and 1 = female) significantly influenced sleep disturbances (*B* = 0.275, *p* < 0.001). Follow‐up sleep disturbances were also predicted by baseline sleep disturbances (*β* = 0.535 *p* < 0.001), age (*β* = −0.150 *p* < 0.001), and having attended psychiatric/psychological support services (*B* = 0.279, *p* < 0.001).

## 4 DISCUSSION

The aim of this study was to test the longitudinal associations between perceived stress during COVID‐19 first wave and sleep disturbances reported at 8–10 months follow‐up. First, we showed that the prevalence of moderate to extremely severe stress symptoms was at 39.4%. This estimate is consistent with rates found in other populations in the same period using the same instrument to assess perceived stress (e.g., Islam et al., 2020; Mazza et al., 2020). Moreover, this finding supports the conceptualization of COVID‐19 pandemic as a life event potentially activating the stress system (Saalwirth & Leipold, 2021; Taylor, 2021). Also, we showed that the prevalence of sleep disturbances during the pandemic (54.8%–57.4%), were in line with other reports (e.g., Cellini et al., 2020; Gualano et al., 2020) and greater than pre‐COVID‐19 estimates (Ohayon & Smirne, 2002).

Second, we showed that stress symptoms during the first pandemic wave was predictive of self‐reported sleep disturbances 8–10 months later, even after controlling for baseline sleep disturbances. This finding is particularly important as previous literature on stress and sleep during COVID‐19 pandemic was limited to cross‐sectional evidence (e.g., Bacaro et al., 2020; Benham, 2020; Franceschini et al., 2020; Saalwirth & Leipold, 2021). Moreover, this finding is consistent with the classical model of neuroendocrine stress response where the release of a cascade of hormones related to the activation of HPA axis (e.g., corticotropin releasing hormone, adrenocorticotropin hormone, and cortisol) is seen to inhibit sleep and promote wakefulness (Cheeta et al., 1997; Lo Martire et al., 2020). Additionally, this finding is in line with the application of the stress‐diathesis model to sleep disturbances which postulates that the occurrence of a stressful life event may act as a precipitating factor of sleep disturbances, triggering the onset of sleep disturbances in already at‐risk individuals (e.g., Spielman et al., 1987).

Findings on mental health showed that depression and anxiety symptoms did not significantly contribute to the onset of sleep disturbances. In the context of sleep medicine, sleep disturbances are usually conceptualized as antecedents of depression and anxiety in meta‐analytic reviews (e.g., Hertenstein et al., 2019). However, there is also robust longitudinal evidence that sleep disturbances and mental health symptoms are bidirectionally linked (e.g., Cox & Olatunji, 2016; Fang et al., 2019). Thus, our finding may sound in contrast with previous literature, yet they may be explained by statistical reasons. Partial regression coefficients consider all the predictors included in the model; thus, given the well‐known high inter‐correlations between the three DASS‐21 scales (Lovibond & Lovibond, 1995), it is not surprising that neither the depression nor the anxiety scales reached the statistical significance. Supporting this interpretation, we found that referring to psychological services predicted greater sleep disturbances; thus, this result indirectly confirms an association between mental health status and sleep disturbances.

With respect to sex, consistent with epidemiological literature (e.g., Suh et al., 2018), we showed that females were most likely to develop sleep disturbances under stress condition. With respect to age, we found that younger individuals were more likely to report sleep disturbances compared to older adults. This finding may sound in contrast with traditional epidemiological literature showing that the prevalence of sleep disturbances increases with age (Ohayon & Smirne, 2002). However, a previous study conducted during COVID‐19 showed that the effect size of pre‐to‐post pandemic change in sleep parameters was moderate for young adults and small for older adults (Sella et al., 2021). In exploratory analysis we showed that younger individuals (those below the median age of 28 years) were also those reporting higher stress symptoms (*F* = 20.938; *p* < 0.0001). Thus, this finding may be plausibly explainable by the greater impact of COVID‐19 in younger versus older individuals. In support of this interpretation, it has been shown that the psychological impact of pandemic‐related restrictions (e.g., increase in loneliness and social isolation) was higher in younger compared to older adults (Beam & Kim, 2020).

Screening positive for COVID‐19 infection and to the death of a strict contact were not significantly involved in the onset of sleep disturbances. This suggests that these factors were unlikely to play a role in the stress‐sleep relationship during the pandemic. Therefore, several mediators and moderators of the stress‐sleep relationship during COVID‐19 remain unknown but could be hypothesized. First, it is possible that classical hormonal response (Lo Martire et al., 2020) may explain the trajectory leading from stress to sleep disturbances. Furthermore, it is possible that the magnitude of the associations between stress and sleep may vary in function of specific moderators. Specifically, pre‐COVID‐19 research showed that sleep reactivity, that is, the trait‐like degree to which individuals exhibit sleep‐disruptive responses to stress, is a possible moderator of the stress‐sleep relationship (Kalmbach et al., 2018). In other words, individuals with higher reactive sleep may experience more pronounced deterioration of sleep during stress exposure, whilst those with lower sleep reactivity may maintain their sleep largely unperturbed during stress (Kalmbach et al., 2018). Also, pre‐COVID‐19 genetic research showed that the expression of serotonin‐transporter‐linked promoter region (5HTTLPR), which affects synaptic serotonin levels, may predispose an individual to experience poor sleep when facing a stressful life event (Harvey et al., 2014).

This study encompasses several limitations that should be acknowledged. First, the MOS scale captures several symptoms of sleep disturbances including sleep initiation, maintenance disturbances, respiratory problems, quantity of sleep, perceived adequacy, and somnolence which may differently be influenced by stress. Since the stress‐diathesis model was particularly applied to explain the onset of insomnia (Perlis et al., 1997; Spielman et al., 1987), future studies on specific and validated insomnia measures (Riemann et al., 2017) would be needed to confirm our findings. Related to this, we included only self‐reported measures of stress and sleep disturbances. Although the definition of several sleep disorders such as insomnia is based on subjective reports (Riemann et al., 2017), the inclusion of objective (polysomnographic) assessment of sleep would have informed about the physiological effects of stress on sleep. Similarly, the inclusion of objective assessment of stress indices (e.g., cortisol, autonomic activity), would have provided information on the specific physiological stress components affecting sleep. Moreover, although this study has the strength to provide longitudinal evidence on the stress‐sleep disturbances association, we included only two assessment points of stress, depression, anxiety, and sleep disturbances; thus, we were unable to test more complex mediation paths between variables under study, and this should be acknowledged as a limitation of the present study. Finally, nearly the 75% of our sample was childless, and this may limit the external validity of the study (e.g., underestimating the impact of stress on sleep for individuals with children in a time of reduced childcare and home‐schooling).

In conclusion, our findings show that COVID‐19 pandemic involve a high prevalence of stress symptoms that may predict the future onset of sleep disturbances. Females and younger individuals may be those at higher risk of developing worse sleep outcomes. Importantly, sleep disturbances are considered risk factors for the onset of future mental and physical conditions including depression, neurodegenerative and inflammatory diseases (Ballesio et al., 2021; Hertenstein et al., 2019; Westhovens et al., 2014). Additionally, it has been shown that the combined effect of perceived stress and poor sleep may account for up nearly 60% of physical health symptoms (Benham, 2009), even after controlling for the effects of negative affect (Benham & Charak, 2018). In line with this consideration, our findings support the importance of psychological stress‐management interventions implementation during the first phases of global health emergencies in order to prevent future onset of sleep disturbances and consequent health‐related conditions (Moreno et al., 2020).

## CONFLICT OF INTEREST

The authors do not report any conflict of interest.

## Data Availability

The data underlying this article will be shared upon reasonable request to the corresponding author.
